# (4*S*,5*S*)-2-(2-Thien­yl)-1,3-dioxolane-4,5-dicarboxamide

**DOI:** 10.1107/S1600536809008368

**Published:** 2009-03-14

**Authors:** Wei Xu, Zheng Yang, Xin-Hua Li, Bo-Nian Liu, De-Cai Wang

**Affiliations:** a State Key Laboratory of Materials-Oriented Chemical Engineering, College of Life Science and Pharmaceutical Engineering, Nanjing University of Technology, Xinmofan Road No. 5 Nanjing, Nanjing 210009, People’s Republic of China; b College of Science, Nanjing University of Technoolgy, Xinmofan Road No. 5 Nanjing, Nanjing 210009, People’s Republic of China

## Abstract

In the title compound, C_9_H_10_N_2_O_4_S, which is an important inter­mediate for the preparation of anti­tumor platinum drugs, the dioxolane ring adopts an envelope conformation with the C atom bonded to the thienyl ring at the flap position. Intra­molecular N—H⋯O and C—H⋯O hydrogen bonds result in the formation of two five-membered rings having envelope conformations. In the crystal structure, inter­molecular N—H⋯O and C—H⋯O hydrogen bonds link the mol­ecules into a three-dimensional network.

## Related literature

For general background, see: Kim *et al.* (1994 ▶); Pandey *et al.* (1997 ▶). For bond-length data, see: Allen *et al.* (1987 ▶).
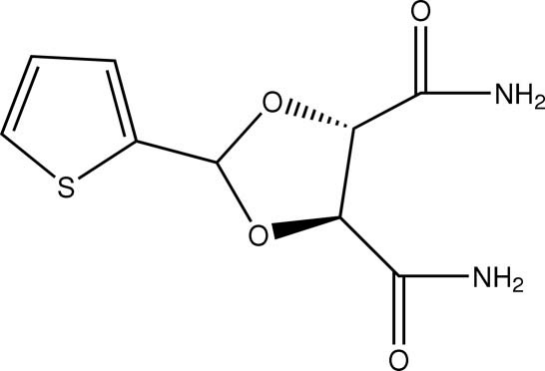


## Experimental

### 

#### Crystal data

C_9_H_10_N_2_O_4_S
*M* *_r_* = 242.25
Monoclinic, 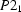

*a* = 8.9250 (18) Å
*b* = 4.796 (1) Å
*c* = 12.109 (2) Å
β = 90.60 (3)°
*V* = 518.29 (18) Å^3^
*Z* = 2
Mo *K*α radiation
μ = 0.31 mm^−1^
*T* = 294 K
0.30 × 0.20 × 0.10 mm

#### Data collection

Enraf–Nonius CAD-4 diffractometer
Absorption correction: ψ scan (North *et al.*, 1968 ▶) *T*_min_ = 0.912, *T*_max_ = 0.969
2135 measured reflections
2007 independent reflections
1820 reflections with *I* > 2σ(*I*)
*R*_int_ = 0.015
3 standard reflections frequency: 120 min intensity decay: 1%

#### Refinement

*R*[*F* ^2^ > 2σ(*F* ^2^)] = 0.040
*wR*(*F* ^2^) = 0.124
*S* = 1.01
2007 reflections
146 parameters
1 restraint
H-atom parameters constrained
Δρ_max_ = 0.25 e Å^−3^
Δρ_min_ = −0.34 e Å^−3^
Absolute structure: Flack (1983 ▶), 876 Friedel pairs
Flack parameter: 0.04 (12)

### 

Data collection: *CAD-4 Software* (Enraf–Nonius, 1989 ▶); cell refinement: *CAD-4 Software*; data reduction: *XCAD4* (Harms & Wocadlo, 1995 ▶); program(s) used to solve structure: *SHELXS97* (Sheldrick, 2008 ▶); program(s) used to refine structure: *SHELXL97* (Sheldrick, 2008 ▶); molecular graphics: *ORTEP-3 for Windows* (Farrugia, 1997 ▶); software used to prepare material for publication: *WinGX* (Farrugia, 1999 ▶).

## Supplementary Material

Crystal structure: contains datablocks global, I. DOI: 10.1107/S1600536809008368/hk2628sup1.cif

Structure factors: contains datablocks I. DOI: 10.1107/S1600536809008368/hk2628Isup2.hkl

Additional supplementary materials:  crystallographic information; 3D view; checkCIF report

## Figures and Tables

**Table 1:** Hydrogen-bond geometry (Å, °)

*D*—H⋯*A*	*D*—H	H⋯*A*	*D*⋯*A*	*D*—H⋯*A*
N1—H1*A*⋯O3^i^	0.86	2.06	2.909 (4)	167
N1—H1*B*⋯O1	0.86	2.30	2.673 (4)	106
N2—H2*A*⋯O3^ii^	0.86	2.29	3.059 (3)	149
N2—H2*B*⋯O4^iii^	0.86	2.28	3.077 (3)	154
C6—H6*A*⋯O4^iii^	0.98	2.27	3.049 (3)	136
C7—H7*A*⋯O4	0.98	2.45	2.866 (3)	105

Symmetry codes: (i) 
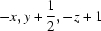
; (ii) 
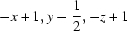
; (iii) 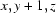.

**Fig. 1.**
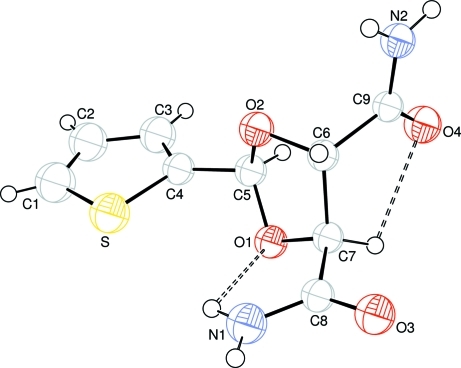
The molecular structure of the title molecule, with the atom-numbering scheme. Hydrogen bonds are shown as dashed lines.

**Fig. 2.**
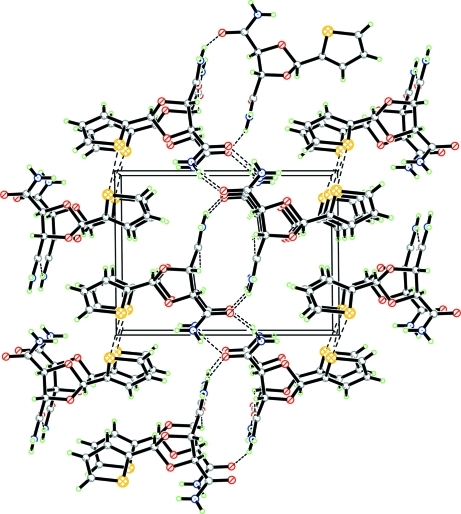
A partial packing diagram of the title compound. Hydrogen bonds are shown as dashed lines.

## supplementary crystallographic information

### Comment

Antitumor platinum drug is one kind of the most effective anticancer agents currently available. (2S,3S)-Diethyl 2,3-O-alkyltartrate analogues are starting materials for the syntheses of platinum complexes with antitumor activity (Kim *et al.*,  1994), and are also important intermediates in organic syntheses (Pandey *et al.*,  1997). As part of our studies on the syntheses and characterizations of these compounds, we have synthesized the title compound and reported herein its crystal structure.

In the molecule of the title compound (Fig 1), the bond lengths (Allen *et al.*,  1987) and angles are within normal ranges. Ring A (S/C1-C4) is, of course, planar, while ring B (O1/O2/C5-C7) adopts an envelope conformation with C5 atom displaced by 0.529 (3) Å from the plane of the other ring atoms. The intramolecular N-H···O and C-H···O hydrogen bonds (Table 1) result in the formations of two five-membered rings C (O1/N1/C7/C8/H1B) and D (O4/C6/C7/C9/H7A), having envelope conformations with atoms O1 and O4 displaced by -0.424 (3) Å and 0.461 (3) Å, respectively, from the planes of the other ring atoms.

In the crystal structure, intermolecular N-H···O and C-H···O hydrogen bonds (Table 1) link the molecules (Fig. 2), in which they may be effective in the stabilization of the structure.

### Experimental

For the preparation of the title compound, a mixture of thiophene-2-carbaldehyde (272 mg, 2.43 mmol), (2S,3S)-diethyltartrate (500 mg, 2.43 mmol), anhydrous copper sulfate (776 mg, 2.86 mmol) and methanesulfonic acid (1 drop) in anhydrous toluene (8 ml) was stirred at room temperature for 12 h. Anhydrous potassium carbonate (40 mg) was added, and then stirred for a further 20 min. The resulting colorless precipitate was obtained by evaporation, and dried in the vacuo. This product (10 mmol) was dissolved in anhydrous ethanol (50 ml), then a current of dry ammonia, dried with calcium chloride, was passed over the reaction mixture at room temperature for about 4 h. The reaction mixture was evaporated to dryness. Crystals suitable for X-ray analysis were obtained by slow evaporation of an ethanol solution after three weeks.

### Refinement

H atoms were positioned geometrically, with N-H = 0.86 Å (for NH_2_) and C-H = 0.93 and 0.98 Å for aromatic and methine H, respectively, and constrained to ride on their parent atoms, with U_iso_(H) = 1.2U_eq_(C,N).

### Crystal data

**Table:**

C_9_H_10_N_2_O_4_S	*F*(000) = 252
*M**_r_* = 242.25	*D*_x_ = 1.552 Mg m^−^^3^
Monoclinic, *P*2_1_	Mo *K*α radiation, λ = 0.71073 Å
Hall symbol: P 2yb	Cell parameters from 25 reflections
*a* = 8.9250 (18) Å	θ = 9–13°
*b* = 4.796 (1) Å	µ = 0.31 mm^−^^1^
*c* = 12.109 (2) Å	*T* = 294 K
β = 90.60 (3)°	Block, colorless
*V* = 518.29 (18) Å^3^	0.30 × 0.20 × 0.10 mm
*Z* = 2	

### Data collection

**Table:**

Enraf–Nonius CAD-4 diffractometer	1820 reflections with *I* > 2σ(*I*)
Radiation source: fine-focus sealed tube	*R*_int_ = 0.015
graphite	θ_max_ = 26.0°, θ_min_ = 1.7°
ω/2θ scans	*h* = 0→10
Absorption correction: ψ scan (North *et al.*, 1968)	*k* = −5→5
*T*_min_ = 0.912, *T*_max_ = 0.969	*l* = −14→14
2135 measured reflections	3 standard reflections every 120 min
2007 independent reflections	intensity decay: 1%

### Refinement

**Table:**

Refinement on *F*^2^	Hydrogen site location: inferred from neighbouring sites
Least-squares matrix: full	H-atom parameters constrained
*R*[*F*^2^ > 2σ(*F*^2^)] = 0.040	*w* = 1/[σ^2^(*F*_o_^2^) + (0.09*P*)^2^] where *P* = (*F*_o_^2^ + 2*F*_c_^2^)/3
*wR*(*F*^2^) = 0.124	(Δ/σ)_max_ < 0.001
*S* = 1.01	Δρ_max_ = 0.25 e Å^−^^3^
2007 reflections	Δρ_min_ = −0.34 e Å^−^^3^
146 parameters	Extinction correction: *SHELXL97* (Sheldrick, 2008), Fc^*^=kFc[1+0.001xFc^2^λ^3^/sin(2θ)]^-1/4^
1 restraint	Extinction coefficient: 0.134 (16)
Primary atom site location: structure-invariant direct methods	Absolute structure: Flack (1983), 876 Friedel pairs
Secondary atom site location: difference Fourier map	Flack parameter: 0.04 (12)

### Special details

**Table:**

Geometry. All e.s.d.'s (except the e.s.d. in the dihedral angle between two l.s. planes) are estimated using the full covariance matrix. The cell e.s.d.'s are taken into account individually in the estimation of e.s.d.'s in distances, angles and torsion angles; correlations between e.s.d.'s in cell parameters are only used when they are defined by crystal symmetry. An approximate (isotropic) treatment of cell e.s.d.'s is used for estimating e.s.d.'s involving l.s. planes.
Refinement. Refinement of *F*^2^ against ALL reflections. The weighted *R*-factor *wR* and goodness of fit *S* are based on *F*^2^, conventional *R*-factors *R* are based on *F*, with *F* set to zero for negative *F*^2^. The threshold expression of *F*^2^ > σ(*F*^2^) is used only for calculating *R*-factors(gt) *etc*. and is not relevant to the choice of reflections for refinement. *R*-factors based on *F*^2^ are statistically about twice as large as those based on *F*, and *R*- factors based on ALL data will be even larger.

### Fractional atomic coordinates and isotropic or equivalent isotropic displacement parameters (Å^2^)

**Table:**

	*x*	*y*	*z*	*U*_iso_*/*U*_eq_	
S	0.14355 (10)	0.3977 (2)	0.03359 (7)	0.0579 (3)	
O1	0.1962 (2)	0.0273 (4)	0.23675 (15)	0.0388 (5)	
O2	0.4057 (2)	0.2850 (4)	0.21945 (16)	0.0409 (5)	
O3	0.1404 (2)	0.2632 (5)	0.51056 (16)	0.0475 (6)	
O4	0.5298 (2)	−0.1909 (4)	0.3765 (2)	0.0496 (6)	
N1	0.0403 (3)	0.4070 (7)	0.3492 (2)	0.0504 (6)	
H1A	−0.0214	0.5180	0.3811	0.060*	
H1B	0.0405	0.3944	0.2784	0.060*	
N2	0.6553 (2)	0.2124 (5)	0.39050 (18)	0.0383 (5)	
H2A	0.7383	0.1315	0.4079	0.046*	
H2B	0.6516	0.3912	0.3856	0.046*	
C1	0.1625 (4)	0.3760 (10)	−0.1056 (3)	0.0639 (10)	
H1C	0.1063	0.4803	−0.1558	0.077*	
C2	0.2684 (4)	0.1899 (10)	−0.1349 (3)	0.0622 (10)	
H2C	0.2952	0.1552	−0.2076	0.075*	
C3	0.3340 (4)	0.0537 (9)	−0.0427 (2)	0.0548 (8)	
H3A	0.4071	−0.0837	−0.0480	0.066*	
C4	0.2777 (3)	0.1472 (6)	0.0543 (2)	0.0397 (7)	
C5	0.3239 (3)	0.0656 (6)	0.1672 (2)	0.0381 (6)	
H5A	0.3843	−0.1049	0.1653	0.046*	
C6	0.3965 (3)	0.2327 (6)	0.3351 (2)	0.0323 (6)	
H6A	0.3940	0.4094	0.3757	0.039*	
C7	0.2451 (3)	0.0773 (6)	0.3475 (2)	0.0329 (6)	
H7A	0.2605	−0.0999	0.3863	0.039*	
C8	0.1346 (3)	0.2558 (6)	0.4091 (2)	0.0347 (6)	
C9	0.5335 (3)	0.0618 (6)	0.3713 (2)	0.0319 (6)	

### Atomic displacement parameters (Å^2^)

**Table:**

	*U*^11^	*U*^22^	*U*^33^	*U*^12^	*U*^13^	*U*^23^
S	0.0688 (6)	0.0563 (5)	0.0484 (4)	0.0176 (4)	0.0005 (4)	0.0024 (4)
O1	0.0370 (10)	0.0429 (11)	0.0365 (10)	−0.0130 (8)	0.0048 (8)	−0.0072 (8)
O2	0.0340 (9)	0.0457 (11)	0.0428 (10)	−0.0131 (8)	−0.0024 (7)	0.0140 (9)
O3	0.0439 (11)	0.0656 (15)	0.0331 (10)	−0.0154 (10)	0.0051 (8)	−0.0026 (10)
O4	0.0446 (11)	0.0252 (11)	0.0788 (16)	−0.0001 (8)	−0.0073 (10)	0.0023 (9)
N1	0.0458 (13)	0.0636 (17)	0.0418 (12)	0.0139 (14)	0.0058 (10)	−0.0047 (15)
N2	0.0315 (11)	0.0310 (12)	0.0523 (14)	−0.0012 (9)	−0.0047 (9)	0.0023 (10)
C1	0.082 (2)	0.066 (2)	0.0434 (17)	−0.002 (2)	−0.0114 (16)	0.0095 (18)
C2	0.065 (2)	0.083 (3)	0.0388 (17)	−0.011 (2)	0.0067 (14)	−0.0020 (17)
C3	0.0546 (18)	0.068 (2)	0.0412 (16)	0.0068 (17)	0.0039 (13)	−0.0059 (16)
C4	0.0343 (13)	0.0417 (16)	0.0432 (15)	−0.0043 (11)	0.0030 (11)	0.0010 (13)
C5	0.0369 (13)	0.0371 (15)	0.0405 (14)	−0.0013 (12)	0.0075 (11)	−0.0011 (12)
C6	0.0313 (12)	0.0255 (12)	0.0402 (14)	−0.0037 (10)	0.0006 (10)	0.0013 (10)
C7	0.0343 (13)	0.0280 (13)	0.0364 (13)	−0.0069 (11)	0.0005 (10)	0.0015 (11)
C8	0.0271 (12)	0.0412 (16)	0.0360 (14)	−0.0115 (11)	0.0017 (10)	−0.0035 (11)
C9	0.0314 (12)	0.0339 (15)	0.0304 (12)	0.0005 (10)	0.0005 (10)	−0.0003 (10)

### Geometric parameters (Å, °)

**Table:**

S—C1	1.698 (3)	C1—C2	1.350 (6)
S—C4	1.713 (3)	C1—H1C	0.9300
O1—C5	1.436 (3)	C2—C3	1.415 (5)
O1—C7	1.426 (3)	C2—H2C	0.9300
O2—C5	1.425 (3)	C3—C4	1.359 (4)
O2—C6	1.426 (3)	C3—H3A	0.9300
O3—C8	1.230 (3)	C4—C5	1.477 (4)
O4—C9	1.214 (4)	C5—H5A	0.9800
N1—C8	1.322 (4)	C6—C7	1.552 (3)
N1—H1A	0.8600	C6—C9	1.532 (4)
N1—H1B	0.8600	C6—H6A	0.9800
N2—C9	1.324 (3)	C7—C8	1.509 (4)
N2—H2A	0.8600	C7—H7A	0.9800
N2—H2B	0.8600		
			
C1—S—C4	91.47 (18)	O2—C5—O1	103.9 (2)
C7—O1—C5	107.04 (19)	O2—C5—C4	110.6 (2)
C5—O2—C6	105.76 (18)	O2—C5—H5A	110.3
C8—N1—H1A	120.0	C4—C5—H5A	110.3
C8—N1—H1B	120.0	O2—C6—C7	103.76 (19)
H1A—N1—H1B	120.0	O2—C6—C9	108.7 (2)
C9—N2—H2A	120.0	O2—C6—H6A	110.0
C9—N2—H2B	120.0	C7—C6—H6A	110.0
H2A—N2—H2B	120.0	C9—C6—C7	114.1 (2)
S—C1—H1C	123.9	C9—C6—H6A	110.0
C2—C1—S	112.3 (3)	O1—C7—C6	104.4 (2)
C2—C1—H1C	123.9	O1—C7—C8	111.4 (2)
C1—C2—C3	112.5 (3)	O1—C7—H7A	110.2
C1—C2—H2C	123.7	C6—C7—H7A	110.2
C3—C2—H2C	123.7	C8—C7—C6	110.5 (2)
C2—C3—H3A	124.0	C8—C7—H7A	110.2
C4—C3—C2	112.1 (3)	O3—C8—N1	123.5 (3)
C4—C3—H3A	124.0	O3—C8—C7	119.3 (2)
C3—C4—S	111.7 (2)	N1—C8—C7	117.1 (2)
C3—C4—C5	127.7 (3)	O4—C9—N2	123.9 (3)
C5—C4—S	120.6 (2)	O4—C9—C6	121.8 (2)
O1—C5—C4	111.2 (2)	N2—C9—C6	114.2 (2)
O1—C5—H5A	110.3		
			
C4—S—C1—C2	−1.2 (3)	S—C4—C5—O2	−69.8 (3)
C1—S—C4—C3	0.2 (3)	C3—C4—C5—O1	−137.8 (3)
C1—S—C4—C5	177.7 (2)	C3—C4—C5—O2	107.3 (4)
C7—O1—C5—O2	−34.8 (2)	O2—C6—C7—O1	7.5 (3)
C7—O1—C5—C4	−153.8 (2)	O2—C6—C7—C8	−112.4 (2)
C5—O1—C7—C8	135.7 (2)	C9—C6—C7—O1	−110.7 (2)
C5—O1—C7—C6	16.5 (3)	C9—C6—C7—C8	129.4 (2)
C6—O2—C5—O1	39.8 (2)	O1—C7—C8—O3	162.6 (2)
C6—O2—C5—C4	159.2 (2)	O1—C7—C8—N1	−20.4 (3)
C5—O2—C6—C7	−28.8 (3)	C6—C7—C8—O3	−81.8 (3)
C5—O2—C6—C9	93.0 (2)	C6—C7—C8—N1	95.1 (3)
S—C1—C2—C3	1.7 (5)	O2—C6—C9—O4	−94.2 (3)
C1—C2—C3—C4	−1.6 (5)	O2—C6—C9—N2	82.7 (3)
C2—C3—C4—S	0.7 (4)	C7—C6—C9—O4	21.0 (4)
C2—C3—C4—C5	−176.6 (3)	C7—C6—C9—N2	−162.1 (2)
S—C4—C5—O1	45.1 (3)		

### Hydrogen-bond geometry (Å, °)

**Table:**

*D*—H···*A*	*D*—H	H···*A*	*D*···*A*	*D*—H···*A*
N1—H1A···O3^i^	0.86	2.06	2.909 (4)	167
N1—H1B···O1	0.86	2.30	2.673 (4)	106
N2—H2A···O3^ii^	0.86	2.29	3.059 (3)	149
N2—H2B···O4^iii^	0.86	2.28	3.077 (3)	154
C6—H6A···O4^iii^	0.98	2.27	3.049 (3)	136
C7—H7A···O4	0.98	2.45	2.866 (3)	105

Symmetry codes: (i) −*x*, *y*+1/2, −*z*+1; (ii) −*x*+1, *y*−1/2, −*z*+1; (iii) *x*, *y*+1, *z*.
